# Variants in *IL23R*-*C1orf141* and *ADO*-*ZNF365*-*EGR2* are associated with susceptibility to Vogt-Koyanagi-Harada disease in Japanese population

**DOI:** 10.1371/journal.pone.0233464

**Published:** 2020-05-21

**Authors:** Takuto Sakono, Akira Meguro, Masaki Takeuchi, Takahiro Yamane, Takeshi Teshigawara, Nobuyoshi Kitaichi, Yukihiro Horie, Kenichi Namba, Shigeaki Ohno, Kumiko Nakao, Taiji Sakamoto, Tsutomu Sakai, Tadashi Nakano, Hiroshi Keino, Annabelle A. Okada, Atsunobu Takeda, Takako Ito, Hisashi Mashimo, Nobuyuki Ohguro, Shinichirou Oono, Hiroshi Enaida, Satoshi Okinami, Nobuyuki Horita, Masao Ota, Nobuhisa Mizuki

**Affiliations:** 1 Department of Ophthalmology and Visual Science, Yokohama City University Graduate School of Medicine, Kanagawa, Japan; 2 Yokosuka Chuoh Eye Clinic, Kanagawa, Japan; 3 Tsurumi Chuoh Eye Clinic, Kanagawa, Japan; 4 Department of Ophthalmology, Health Sciences University of Hokkaido, Hokkaido, Japan; 5 Department of Ophthalmology, Faculty of Medicine and Graduate School of Medicine, Hokkaido University, Hokkaido, Japan; 6 Department of Ophthalmology, Kagoshima University Graduate School of Medical and Dental Sciences, Kagoshima, Japan; 7 Department of Ophthalmology, Jikei University School of Medicine, Tokyo, Japan; 8 Department of Ophthalmology, Kyorin University School of Medicine, Tokyo, Japan; 9 Department of Ophthalmology, Graduate School of Medical Sciences, Kyushu University, Fukuoka, Japan; 10 Department of Ophthalmology, Japan Community Health care Organization Osaka Hospital, Osaka, Japan; 11 Department of Ophthalmology, Saga University Faculty of Medicine, Saga, Japan; 12 Hoshiai Eye Clinic, Saitama, Japan; 13 Department of Ophthalmology, Kurashiki Central Hospital, Okayama, Japan; 14 Department of Pulmonology, Yokohama City University Graduate School of Medicine, Kanagawa, Japan; 15 Division of Hepatology and Gastroenterology, Department of Medicine, Shinshu University School of Medicine, Nagano, Japan; Kunming Institute of Zoology, Chinese Academy of Sciences, CHINA

## Abstract

Vogt-Koyanagi-Harada (VKH) disease is a systemic inflammatory disorder that affects pigment cell-containing organs such as the eye (e.g., chronic and/or recurrent granulomatous panuveitis). While the exact etiology and pathogenic mechanism of VKH disease are unclear, *HLA-DR4* alleles have been documented to be strongly associated with VKH disease in various ethnic groups. Recently, a genome-wide association study (GWAS) found two new genetic risk factors (*IL23R-C1orf141* and *ADO-ZNF365-EGR2*) in a non-*HLA* region from a Han Chinese population. In this study, we replicated these GWAS findings in a Japanese population. A total of 1,643 Japanese samples (380 cases with VKH disease and 1,263 healthy controls) were recruited. We assessed four single nucleotide polymorphisms (SNPs) shown in previous GWAS: rs78377598 and rs117633859 in *IL23R-C1orf141*, and rs442309 and rs224058 in *ADO-ZNF365-EGR2*. A significant allelic association with VKH disease was observed for all of the four SNPs (rs78377598: p_c_ = 0.0057; rs117633859: p_c_ = 0.0017; rs442309: p_c_ = 0.021; rs224058: p_c_ = 0.035). In genotypic association analysis, the minor alleles of *IL23R-C1orf141* rs78377598 and rs117633859 had the strongest association with disease susceptibility under the additive model (p_c_ = 0.0075 and p_c_ = 0.0026, respectively). The minor alleles of *ADO-ZNF365-EGR2* rs442309 and rs224058 were most strongly associated with disease susceptibility under the dominant model (p_c_ = 0.00099 and p_c_ = 0.0023, respectively). The meta-analysis of the current and previous studies found that all of the four SNPs exhibited a significantly strong association with VKH disease (meta-p < 0.00001: rs78377598, meta-odds ratio (OR) = 1.69; rs1176338, meta-OR = 1.82; rs442309, meta-OR = 1.34; rs224058, meta-OR = 1.33). In summary, our study replicated significant associations with VKH disease susceptibility reported in a previous GWAS. Thus, the *IL23R-C1orf141* and *ADO-ZNF365-EGR2* loci may play important roles in the development of VKH disease through genetic polymorphisms.

## Introduction

VKH disease is a systemic polymorphic autoimmune disorder that targets organs with melanocytes such as the eye, meninges, inner ear, skin, and hair [1]. VKH disease, along with sarcoidosis and Behcet’s disease, is one of the causes of endogenous uveitis and an ophthalmological condition that is most common in the Japanese population [2,3]. VKH disease in the acute stage is characterized by the development of bilateral uveitis associated with multifocal exudative retinal detachment (RD) in the posterior pole and inflammation signs often observed in the anterior ocular, such as mutton-fat keratic precipitates, iris nodules, and shallow anterior chamber. Early-phase of fluorescein angiography (FA) in the acute stage shows multiple focal areas of leakage at the level of retinal pigment epithelium, and late-phase of FA shows dye pooling within subretinal fluid (SRF). In the chronic stage of VKH disease, sunset glow fundus characterized by orange-red discoloration due to depigmentation of the choroid is found [4–6]. The incidence of VKH disease varies worldwide. The disease occurs more frequently among people with dark skin pigmentation, as well as in those of Asian descent, Native Americans, and Hispanics compared to Caucasians [4,7]. In Japan, VKH disease accounts for about 7% of all uveitis patients [3]. In contrast, VKH disease patients represent only about 1% to 4% of all uveitis cases in the United States [1].

Although the exact etiology of VKH disease remains unclear, genetic factors may play an important role in disease development. A strong association of VKH disease with human leukocyte antigen (*HLA*)*-DR4* has been reported by some ethnic groups [8–11]. The pathogenesis of VKH disease may be implicated by multifactorial factors through environmental triggers and susceptibility genes such as *HLA* and non-*HLA* [12,13].

The characteristic clinical findings of tissue depigmentation in VKH disease point to the possible involvement of melanocytes in the pathogenesis. The tyrosinase gene family (e.g., *tyrosinase*, *tyrosinase-related protein* (*TRP*) *1*, *TRP2* and *dopachrome tautomerase*) is expressed specifically in melanocytes and involve in pigmentation. In earlier studies, *TRP1* and *TRP2* induced an experimental autoimmune disease in Lewis rats. The clinical course and histological findings resembled human VKH disease [14]. It is also reported that human VKH-like disease is induced in Akita dogs by immunizing them with *TRP1* [15]. Further, lymphocytes obtained from VKH disease patients were reactive to peptides derived from tyrosinase gene family [16]. These studies suggest that tyrosinase gene family may be responsible for human VKH disease. However, the association of VKH disease with genes in the tyrosinase gene family has been showed negative results in Japanese patients with VKH disease [13].

A recent genome-wide association study (GWAS) of patients with VKH disease from a Han Chinese population identified two new non-*HLA* candidate regions, namely interleukin 23 receptor (*IL23R*)-chromosome 1 open reading frame 141 (*C1orf141*) on 1p31.2 and 2-aminoethanethiol dioxygenase (*ADO*)-zinc finger protein 365 (*ZNF365*)-early growth response 2 (*EGR2*) on 10q21.3 [17]. These two new loci were also assessed in replication studies that included the Han Chinese in Singapore, a non-Han Chinese population in southwestern China, and patients with VKH disease from Thailand and Korea. In these studies, *IL23R-C1orf141* on 1q31.2 was associated with VKH disease among patients of Han Chinese descent in Singapore but not in those of other Asian ethnicities. The association between *ADO-ZNF365-EGR2* on 10q21.3 and VKH disease has only been confirmed in a Thai population [18].

To further explore these issues, we conducted a replication study in Japanese patients with VKH disease. We investigated an association between VKH disease and four single nucleotide polymorphisms (SNPs), namely rs78377598 and rs117633859 on 1p31.2 and rs1142309 and rs224058 on 10q21.3, which have been previously reported [17,18]. In addition, we performed a random-effects meta-analysis of the odds ratios (ORs) of four SNPs in Japanese and other Asian populations.

## Materials and methods

### Participants

We recruited 380 unrelated Japanese patients with VKH disease (41.6% male, mean age 51.3 ± 14.7 years [range 21 to 81 years]) and 1,263 unrelated Japanese healthy controls (46.8% male, mean age 54.6 ± 14.3 years [range 20 to 87 years]) (Table 1). The patients were diagnosed between 2003 and 2015 according to the “Revised Diagnostic Criteria for VKH Disease” at the Uveitis Survey Clinic of Yokohama City University, Hokkaido University, Kagoshima University, Jikei University, Kyorin University, Kyusyu University, Japan Community Healthcare Organization Osaka Hospital, and Saga University. All patients met the criteria established by the 2001 First International Workshop on Vogt-Koyanagi-Harada Disease [9]. The details of criteria are (i) no history of penetrating ocular trauma / surgery before uveitis onset, (ii) no clinical / laboratory evidence of other ocular disease, (iii) bilateral ocular involvement: diffuse choroiditis (focal regions of SRF, bullous serous RD) and FA (focal areas of delay in choroidal perfusion, multifocal areas of hyperfluorescence, pooling within SRF, and optic nerve staining), (iv) cerebrospinal fluid pleocytosis, and (v) integumentary findings (alopecia, poliosis and vitiligo) [19]. Clinical presentation showed little variation among patients. The control subjects were all healthy volunteers of similar ethnic origin as the patients, and were not related to each other or to the VKH disease patients. All controls had no clinical manifestations or family history of any type of immune-related diseases. All participants gave their written informed consent. The study was approved by the ethics committees of Yokohama City University, Hokkaido University, Kagoshima University, Jikei University, Kyorin University, Kyusyu University, Japan Community Healthcare Organization Osaka Hospital, and Saga University and conducted in accordance with the Declaration of Helsinki and its subsequent revisions.

**Table 1:** Characteristics of the study populations.

Characteristic	Cases (n = 380)	Controls (n = 1,263)
Male	41.6%	46.8%
Mean age [SD; range], years	51.3 [14.7; 21–81]	54.6 [14.3; 20–87]
Uveitis	100.0%	
Nuchal rigidity	10.9%	
Headache	58.4%	
Scalp allergy	11.9%	
Tinnitus	32.2%	
Dysacusia	22.3%	
Alopecia	6.9%	
Poliosis	10.4%	
Vitiligo	6.4%	

SD, standard deviation.

### SNP genotyping within *IL23R-C1orf141* and *ADO-ZNF365-EGR2* genes

We assessed the four SNPs that showed a strong association with VKH disease in a previous GWAS: rs78377598 and rs117633859 in *IL23R-C1orf141* on the 1p31.2 locus, and rs442309 and rs224058 in *ADO-ZNF365-EGR2* on the 10q21.3 locus [17]. Genomic DNA was extracted from peripheral blood samples using the QIAamp DNA Blood Mini Kit (Qiagen, Hilden, Germany). Standardized conditions were used to prevent variation in DNA quality. SNP genotyping was performed using the TaqMan 5' exonuclease assay with primers supplied by Applied Biosystems (Foster City, CA, USA). Polymerase chain reaction (PCR) was performed in a 10 μL reaction mixture containing 1× TaqMan Universal PCR Master Mix (Applied Biosystems), 24 nm of each primer-probe set, and 3 ng genomic DNA. The PCR conditions were as follows: 95°C for 10 min, followed by 40 cycles of denaturation at 92°C for 15 s and annealing/extension at 60°C for 1 min. The probe’s fluorescence signal was detected using the StepOnePlus Real-Time PCR System (Applied Biosystems).

### Statistical analysis

We performed allelic and genotypic association analyses, and calculated Hardy-Weinberg equilibrium using SNP and Variation Suite 8.4.0 software (Golden Helix, Inc., Bozeman, MT, USA, http://www.goldenhelix.com). For genotypic association analysis, we applied three different genetic models to assess each minor allele: additive (2/2 vs. 1/2 vs. 1/1), dominant (2/2+1/2 vs. 1/1), and recessive (2/2 vs. 1/2+1/1) models (assuming that 2 is the minor allele and 1 is the major allele). Differences in allele and genotype frequencies between cases and controls were assessed by correlation/trend test. The p-values and ORs in genotype models were adjusted for age and sex. The obtained p-values were corrected for multiple testing using Bonferroni’s method based on the number of tested SNPs (n = 4). A corrected p-value (pc) < 0.05 was considered significant.

### Random-effects meta-analysis

We conducted a random-effects meta-analysis of the current and previous studies using the generic inverse variance method and logarized OR. The pooled OR corresponding to one risk allele increase in allelic model for each SNP was calculated [20–22]. The heterogeneity was estimated using I^2^ statistics as follows: 0%: indicates no heterogeneity; 0% to 30%: might not be important; 30% to 50%: may represent moderate heterogeneity; 50% to 75%: may represent substantial heterogeneity; 75% to 100%: considerable heterogeneity [22]. We used Review Manager ver. 5.3 (Cochrane Collaboration, Oxford, UK) to perform meta-analysis.

## Results

We performed genotyping of four SNPs in the VKH disease patient and control groups. The genotype frequencies of all four SNPs were all in Hardy-Weinberg equlibirum for the cases and controls. Table 2 shows the allelic association results for the four SNPs. Two SNPs (rs78377598 and rs117633859) in *IL23R*-*C1orf141* were significantly associated with VKH disease in the Japanese population (p = 0.0014 and p = 0.00043, respectively). Statistical significance was kept following Bonferroni's correction (rs78377598: p_c_ = 0.0057 and rs117633859: p_c_ = 0.0017). The T allele of rs78377598 and the G allele of rs117633859 were more frequent alleles in patients with VKH disease than the control group, indicating that these alleles were susceptible to VKH disease (OR = 1.65 and OR = 1.71, respectively). The variants in the *ADO-ZNF365-EGR2* locus also showed a significant association with the disease (rs442309: p = 0.0053, p_c_ = 0.021 and rs224058: p = 0.0088, p_c_ = 0.035). Both allele frequencies of the T allele of rs442309 and the A allele of rs224058 were higher in VKH disease patients compared to the controls (OR = 1.27 and OR = 1.25, respectively).

**Table 2:** Allelic association results for rs78377598 and rs117633859 in *IL23R-C1orf141*, and rs442309 and rs224058 in *ADO-ZNF365-EGR2*.

					Minor Allele Freq., %				
SNP	Chr.	Position (GRCh37)	Alleles (1>2)	Call Rate, %	Cases (n = 380)	Controls (n = 1,263)	*P*	*P*c	OR (95% CI)
*IL23R-C1orf141*									
rs78377598	1	67,612,502	C>T	98.8	8.4	5.3	0.0014	0.0057	1.65 (1.21–2.25)
rs117633859	1	67,627,828	A>G	98.9	9.1	5.5	0.00043	0.0017	1.71 (1.26–2.31)
*ADO-ZNF365-EGR2*									
rs442309	10	64,490,495	C>T	99.1	42.8	37.2	0.0053	0.021	1.27 (1.07–1.49)
rs224058	10	64,498,865	G>A	98.8	42.6	37.3	0.0088	0.035	1.25 (1.06–1.47)

1, major allele; 2, minor allele; OR, odds ratio; CI, confidence interval.

The results of genotypic association analysis for the four SNPs under different genetic models are presented in Table 3. The minor alleles of rs78377598 and rs117633859 in *IL23R-C1orf141* had the strongest association with the risk of VKH disease under the additive model (rs78377598: p = 0.0019, p_c_ = 0.0075, OR = 1.64 and rs117633859: p = 0.00066, p_c_ = 0.0026, OR = 1.70). These alleles were also significantly associated with VKH disease under the dominant model (rs78377598: p = 0.0031, p_c_ = 0.012, OR = 1.68 and rs117633859: p = 0.0011, p_c_ = 0.0043, OR = 1.75). The minor alleles of rs442309 and rs224058 in *ADO-ZNF365-EGR2* were most strongly associated with the risk of VKH disease under the dominant model (rs442309: p = 0.00025, p_c_ = 0.00099, OR = 1.58 and rs224058: p = 0.00057, p_c_ = 0.0023, OR = 1.53) and also showed a significant association with the disease under the additive model (rs442309: p = 0.0061, p_c_ = 0.025, OR = 1.26 and rs224058: p = 0.0092, p_c_ = 0.037, OR = 1.24). No significant association was found for any of the four SNPs in the recessive model (p > 0.05).

**Table 3:** Genotypic association results for rs78377598 and rs117633859 in *IL23R-C1orf141*, and rs442309 and rs224058 in *ADO-ZNF365-EGR2*.

		Genotype ((2/2)/(1/2)/(1/1)) Frequency, %		-Genetic Models^a^							
SNP	Alleles (1>2)	Cases (n = 380)	Controls (n = 1,263)	Additive (2/2 vs. 1/2 vs. 1/1)			Dominant (2/2+1/2 vs. 1/1)			Recessive (2/2 vs. 1/2+1/1)	
				*P*	*P*c	OR (95% CI)	*P*	*P*c	OR (95% CI)	P	OR (95% CI)
*IL23R-C1orf141*											
rs78377598	C>T	1.3/14.2/84.5	0.5/9.6/89.9	0.0019	0.0075	1.64 (1.21–2.23)	0.0031	0.012	1.68 (1.20–2.35)	0.10	2.84 (0.85–9.42)
rs117633859	A>G	1.3/15.5/83.2	0.5/10.1/89.4	0.00066	0.0026	1.70 (1.26–2.29)	0.0011	0.0043	1.75 (1.26–2.42)	0.090	2.94 (0.88–9.80)
*ADO-ZNF365-EGR2*											
rs442309	C>T	16.5/52.5/30.9	15.6/43.2/41.3	0.0061	0.025	1.26 (1.07–1.48)	0.00025	0.00099	1.58 (1.23–2.02)	0.65	1.08 (0.78–1.47)
rs224058	G>A	16.5/52.1/31.4	15.5/43.5/41.0	0.0092	0.037	1.24 (1.06–1.47)	0.00057	0.0023	1.53 (1.20–1.96)	0.64	1.08 (0.79–1.48)

1, major allele; 2, minor allele; OR, odds ratio; CI, confidence interval.

^a^p-values and ORs were adjusted for age and sex.

**Fig 1:**
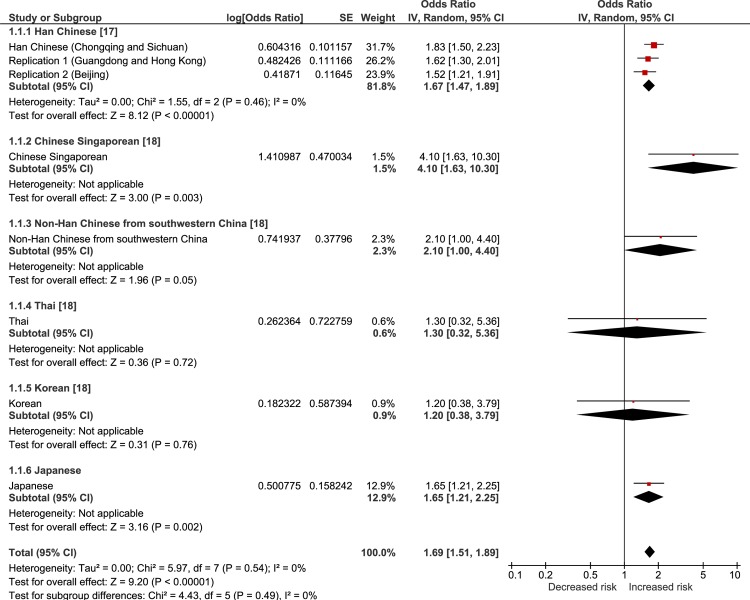
Forest plot of the meta-analysis of the association of *IL23R-C1orf141* rs78377598 and VKH disease. The lines with squares in the middle correspond to the study-specific 95% CI and OR. The central vertical solid line indicates the OR for the null hypothesis. The diamond represents the summary OR with its corresponding 95% CI.

**Fig 2:**
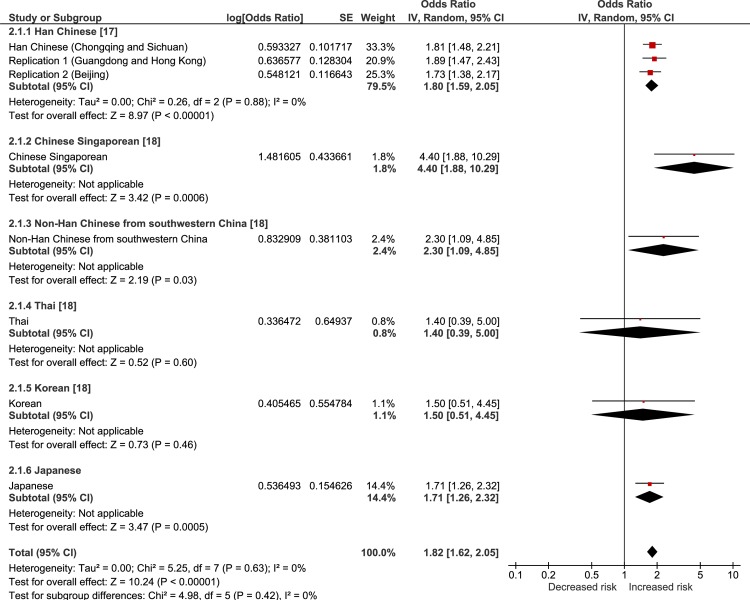
Forest plot of the meta-analysis of the association of *IL23R-C1orf141* rs1176338 and VKH disease. The lines with squares in the middle correspond to the study-specific 95% CI and OR. The central vertical solid line indicates the OR for the null hypothesis. The diamond represents the summary OR with its corresponding 95% CI.

**Fig 3:**
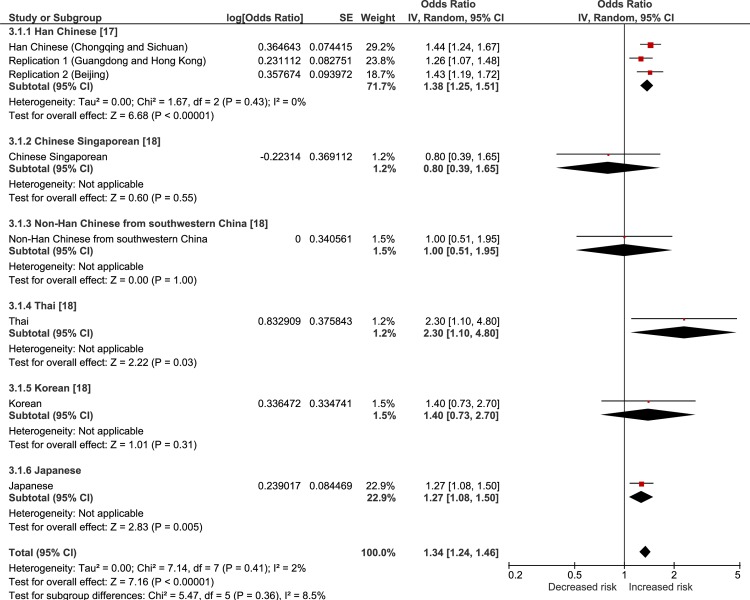
Forest plot of the meta-analysis of the association of *ADO-ZNF365-EGR2* rs442309 and VKH disease. The lines with squares in the middle correspond to the study-specific 95% CI and OR. The central vertical solid line indicates the OR for the null hypothesis. The diamond represents the summary OR with its corresponding 95% CI.

**Fig 4:**
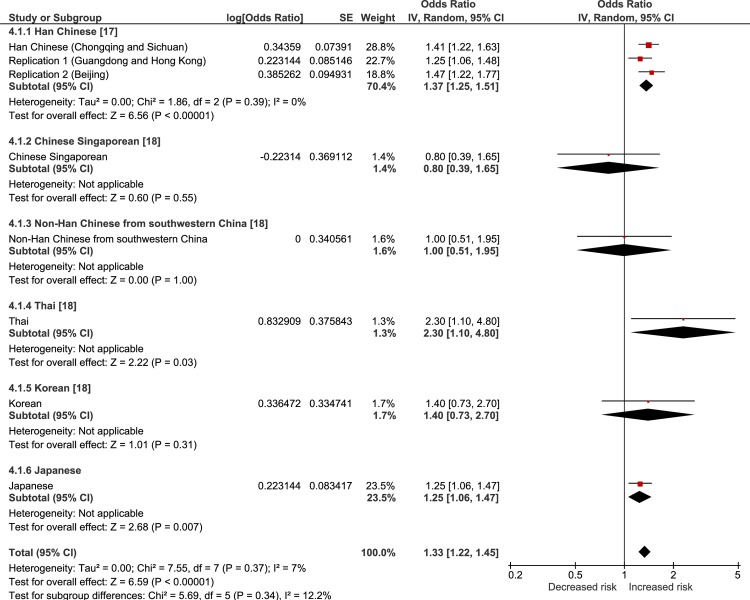
Forest plot of the meta-analysis of the association of *ADO-ZNF365-EGR2* rs224058 and VKH disease. The lines with squares in the middle correspond to the study-specific 95% CI and OR. The central vertical solid line indicates the OR for the null hypothesis. The diamond represents the summary OR with its corresponding 95% CI.

The results of a random-effects meta-analysis of the current and previous studies are showed in Figs 1–4. The meta-analysis revealed that all of the tested SNPs exhibited a significantly strong association with the risk of VKH disease (meta-p < 0.00001, rs78377598: meta-OR = 1.69; rs117633859: meta-OR = 1.82; rs442309: meta-OR = 1.34; rs224058: meta-OR = 1.33). rs78377598 and rs1176338 in *IL23R-C1orf141* had an increased risk of VKH disease in all ethnic populations (Figs 1 and 2). On the other hand, rs442309 and rs224058 in *ADO-ZNF365-EGR2* did not always have an increased risk in all popuations (OR ≤ 1.0 in the Han Chinese group in Singapore and the non-Han Chinese group) (Figs 3 and 4).

## Discussion

VKH disease is a systemic autoimmune inflammatory disorder. A number of studies have shown that genetic polymorphisms including *HLA* genes and non-*HLA* genes affect the susceptibility of VKH disease. Hou et al. identified new non-*HLA* candidate genes, namely *IL23R-C1orf141* on 1p31.2 and *ADO-ZNF365-EGR2* on 10q21.3, by GWAS targeting a group of Han Chinese patients with VKH disease [17]. In this study, we also found that these two genes are susceptibility genes involved in the pathogenesis of VKH disease in Japanese patients.

A previous report showed that the interleukin 23 receptor (*IL23R*) is expressed in the iris and ciliary bodies of healthy subjects, while *C1orf141* is only expressed in the iris of healthy subjects [17]. Furthermore, genetic variants in *IL23R* are associated with multiple immune-related diseases such as Behcet’s disease, Crohn’s disease, ulcerative colitis, psoriasis, and ankylosing spondylitis [23–31]. *IL23R* is expressed in type 17 helper T cells (Th17) cells, which are implicated in the pathogenesis of various immune-mediated diseases. *IL23* signaling through the *IL23R* promotes the proliferation, maintenance, and activation of Th17 inducing neutrophil inflammation and autoimmune diseases [32–34]. Liang et al. reported that VKH disease patients with active uveitis had significantly higher percentages of Th17 and IL-23 as compared with inactive VKH disease patients and healthy controls [35]. *C1orf141* is involved in psoriasis [36]; however, its function remains to be elucidated. These reports indicate that an activation of Th17 through the *IL23R* is involved in the pathogenesis of VKH disease. In the current study, we confirmed the significant association of rs78377598 and rs117633859 in *IL23R-C1orf141* with VKH disease in the Japanese popualtion. Taken together, *IL23R* are likely involved in the development of VKH disease through genetic variants of *IL23R-C1orf141*.

In this study, we succeeded in replicating previous GWAS findings showing that *ADO-ZNF365-EGR2* is susceptibility locus involved in the development of VKH disease in the Japanese population. However, these findings were not reproduced in the Han Chinese Singaporeans and, the non-Han Chinese population from southwestern China [18]. This difference in results may be due to the small sample size in the previous replication study, which may increase the risk of false negative results, called type II errors. Our study recruited 380 Japanese patients with VKH disease. In contrast, the previous study was conducted with 32 cases from the Han Chinese Singaporeans, 38 cases from a non-Han Chinese population in southwestern China, 81 cases from Thai, and 34 cases from Koreans [18]. Obviously, these sample sizes were not enough to produce effective statistical results: hence, it is suggested that the previous study could not detect the association between VHK disease and *ADO-ZNF365-EGR2* in the Han Chinese Singaporeans and the non-Han Chinese. In addition, it has been hypothesized that VKH disease may be triggered by virus infections, such as the Epstein-Barr virus and the cytomegalovirus [37,38]. This hypothesized factors may be dependent on environmental factors, Asian countries have different environmental factors [39]. Therefore, the etiology and disease mechanisms underlying VKH disease development may be elucidated by the effects of gene-environment interactions. *ADO*, *ZNF365*, and *EGR2* are expressed in the iris and *EGR2* is expressed in ciliary bodies and the choroid [18]. Moreover, these genes are reportedly associated with multiple immune-related diseases such as Behcet’s disease, Crohn’s disease, ulcerative colitis, atopic dermatitis, and systemic lupus erythematosus [25,26,40–44]. Thus, *ADO-ZNF365-EGR2* may play suggestive effects in pathogenesis and mechanism of VKH disease in Japanese patients.

There were some differences in the disease-risk allele frequencies of *IL23R-C1orf141* and *ADO-ZNF365-EGR2* among control groups of Asian populations used in the current and previous studies (*IL23R-C1orf141* rs78377598 and rs117633859: 4.5–5.9% in Japanese, Han Chinese Singaporeans [18], Thai [18], and Koreans [18], 9.5–9.7% in Han Chinese [17], 13.9–14.2% in non-Han Chinese from southwestern China [18]; *ADO-ZNF365-EGR2* rs442309 and rs224058: 37.2–37.3% in Japanese, 25.6% in Han Chinese [17], 22.3% in Han Chinese Singaporeans [18], 30.8% in non-Han Chinese from southwestern China [18], 18.2% in Thai [18], 33.0% in Koreans [18]). However, the differences did not reflect the differences in association between VKH disease and the two loci among the Asian populations. The disease-risk allele frequencies of *IL23R-C1orf141* are lower in Caucasians (2.5% in Caucasians from 1000 Genomes Project Phase 3 [45]) that have a low prevalence of VKH disease than in Asians, and those of *ADO-ZNF365-EGR2* in Caucasians (52.0% [45]) are higher in Asians.

In conclusion, the current study confirmed a significant association between VKH disease and the two loci, *IL23R-C1orf141* and *ADO-ZNF365-EGR2*, in the Japanese population, suggesting that genetic variants in these loci play important roles in disease development. To confirm and validate the correlation between VKH disease and these loci, future genetic studies with larger samples of Asian and other ethnic populatitons are needed.
